# Isolation and Epitope Mapping of Staphylococcal Enterotoxin B Single-Domain Antibodies

**DOI:** 10.3390/s140610846

**Published:** 2014-06-19

**Authors:** Kendrick B. Turner, Dan Zabetakis, Patricia Legler, Ellen R. Goldman, George P. Anderson

**Affiliations:** 1 American Society for Engineering Education, Postdoctoral Fellow at the Naval Research Laboratory, Washington, DC 20375, USA; E-Mail: kendrick.turner.ctr@nrl.navy.mil; 2 Center for Biomolecular Science and Engineering, Naval Research Laboratory, Washington, DC 20375, USA; E-Mails: daniel.zabetakis@nrl.navy.mil (D.Z.); patricia.legler@nrl.navy.mil (P.L.); ellen.goldman@nrl.navy.mil (E.R.G.)

**Keywords:** nanobodies, surface plasmon resonance, circular dichroism

## Abstract

Single-domain antibodies (sdAbs), derived from the heavy chain only antibodies found in camelids such as llamas have the potential to provide rugged detection reagents with high affinities, and the ability to refold after denaturation. We have isolated and characterized sdAbs specific to staphylococcal enterotoxin B (SEB) which bind to two distinct epitopes and are able to function in a sandwich immunoassay for toxin detection. Characterization of these sdAbs revealed that each exhibited nanomolar binding affinities or better. Melting temperatures for the sdAbs ranged from approximately 60 °C to over 70 °C, with each demonstrating at least partial refolding after denaturation and several were able to completely refold. A first set of sdAbs was isolated by panning the library using adsorbed antigen, all of which recognized the same epitope on SEB. Epitope mapping suggested that these sdAbs bind to a particular fragment of SEB (VKSIDQFLYFDLIYSI) containing position L45 (underlined), which is involved in binding to the major histocompatibility complex (MHC). Differences in the binding affinities of the sdAbs to SEB and a less-toxic vaccine immunogen, SEBv (L45R/Y89A/Y94A) were also consistent with binding to this epitope. A sandwich panning strategy was utilized to isolate sdAbs which bind a second epitope. This epitope differed from the initial one obtained or from that recognized by previously isolated anti-SEB sdAb A3. Using SEB-toxin spiked milk we demonstrated that these newly isolated sdAbs could be utilized in sandwich-assays with each other, A3, and with various monoclonal antibodies.

## 1. Introduction

*Staphylococcus aureus*, a common pathogenic bacteria, produces several super antigenic virulence factors known as staphylococcal enterotoxins [1,2]. Of these heat-resistant enterotoxins, staphylococcal enterotoxin B (SEB), a 28 kDa protein consisting of 239 amino acids, has been of particular interest because it is one of the most common causes of foodborne illnesses, toxic shock syndrome, and a potential bioterrorism and biowarfare threat [3,4]. The structure of SEB consists of two distinct domains including an N-terminal, β-barrel-like domain and a C-terminal domain rich in α-helices and containing a β-grasp motif. The toxicity of SEB is mediated through its interaction with the major histocompatibility complex (MHC) class II on target cells resulting in widespread proliferation of leukocytes and cytokine release. SEB can be isolated in 50%–80% of *S. aureus* strains including those which are methicillin resistant (MRSA) [5,6]. SEB exotoxins are potential biowarfare agents because they are highly stable, cause severe systemic effects (LD_50_ = 0.02 μg/kg), and can be readily aerosolized or propagated in bacteria [7]. Because of these concerns, the development of sensitive and selective detection methods for SEB is of vital importance.

To that end, there have been recent efforts to develop sensing platforms for SEB including photonic crystal-based lab-on-a-chip assays [8], fluorescence-based flow cytometry assays [9], and aptamer-based fluorescence resonance energy transfer (FRET) assays [10]. Each of these approaches requires sensitive and selective recognition elements such as monoclonal antibodies (mAbs), aptamers, or receptors specific to SEB able to bind the toxin in complex sample matrices and elicit a measurable response. Antibodies are commonly used as recognition elements in assays primarily due to their exceptional specificity and nanomolar or lower dissociation constants.

While conventional antibodies have been widely used immunoassay reagents, their complex structure consisting of multiple domains, disulfides, and glycosylations increases the cost and difficulty of their production. Their stability can limit their effectiveness in non-ideal assay conditions (e.g., high temperature or denaturing conditions). As an alternative to traditional antibodies, various antibody fragments have been explored including single-chain variable fragments (scFvs) comprised of the variable regions of conventional antibodies [11,12] and single-domain antibodies (sdAbs) consisting of the variable domain of heavy-chain only antibodies (HCAbs) [13,14]. Several species produce naturally occurring HCAbs including dromedaries (camels, llama, and alpacas) and cartilaginous fishes [15,16]. Their small size, ease of expression in *Escherichia coli*, thermal stability, and ability to refold after denaturation have made them attractive alternatives to conventional antibodies [17]. We previously isolated an sdAb specific for SEB, however to enable sdAb-based sandwich immunoassays for SEB additional binders are required that recognized a different epitope [18]. In this work, we describe the isolation and characterization of additional sdAbs and the development of assays that utilize the newly isolated sdAbs, our previously characterized sdAb A3, and mAbs for the sensitive detection of SEB in complex matrices.

## 2. Results

### 2.1. Evaluation of Serum Anti-SEB Titer

With the goal of identifying additional SEB binding sdAbs, two llamas were immunized, one (Centavo) with SEB toxoid and one (Whisper) with SEBv, a triple mutant developed as a vaccine immunogen [19] following the immunization protocol as described in the Methods section. At the end of the immunization protocol, blood was collected, and the plasma fraction analyzed for SEB-binding antibodies. Results of this anti-SEB titer (not shown) were indicative of a significant immune response against the SEB toxoid in Centavo. In contrast, the anti-SEB titer results from Whisper indicated that a weaker immune response was obtained upon immunization with the SEBv vaccine. As a result, subsequent preparation of a phage display library of SEB-binding sdAbs was constructed using only mRNA derived from the animal immunized with SEB toxoid.

### 2.2. Library Quality and Diversity

To develop a library of anti-SEB sdAbs, mRNA was purified from peripheral blood lymphocytes isolated from blood drawn from the SEB toxoid immunized llama and a cDNA library of sdAbs was cloned into a phage-display vector, pECAN21. A small-scale test ligation and subsequent sequence analysis of 10 clones from each ligation confirmed the presence of unique, in-frame sdAb sequences. A large scale ligation reaction for each library was then performed, and the resulting plasmid library transformed by electroporation into XL1-Blue cells. The final library size was estimated by colony count to be ∼10^8^.

### 2.3. Library Panning

**Figure 1.**
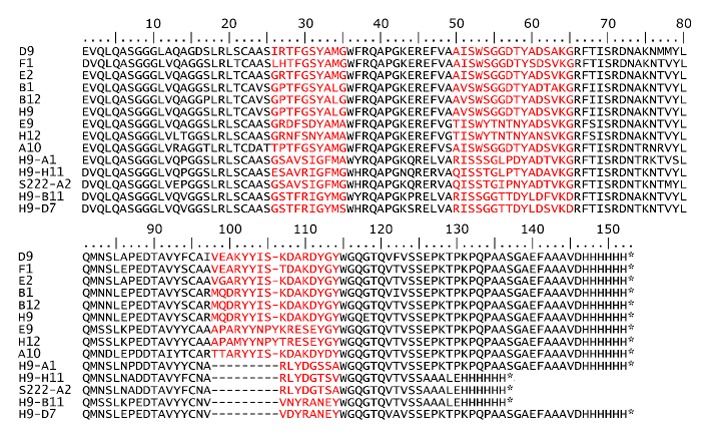
Sequences of isolated SEB binders. Complementarity determining regions (CDRs) have been colored red for clarity. The sequences of three of the sdAbs (H9-H11, S222-A2, and H9-B1) are shorter as they are shown lacking sequence from the upper hinge region.

An initial phage display library was constructed by phage amplification using M13K07 helper phage and subjected to two rounds of panning, with an increased number of washes in each round. Polyclonal ELISA following each round of panning demonstrated a significant enrichment of SEB binders, and the output phage titer increased from 2.5 × 10^6^ cfu/mL to 4.5 × 10^7^ cfu/mL between rounds of panning. Monoclonal ELISA of 48 colonies from each round of panning resulted in 17 positive binders from round one and 25 positive binders from round two. Positive binders were defined as those which resulted in an absorbance signal of greater than 0.25 during monoclonal ELISA. From these binders, ten were sequenced resulting in nine unique sdAb sequences which could be divided into two families based upon sequence homology of the CDRs (Figure 1). E9 and H12 possess similar CDR sequences (especially the beginning of CDR2 and throughout CDR3) and a CDR3 one amino acid longer than the remaining seven sequences. The others have a similar sequence for each of their CDRs.

As the initial panning protocol based on subsequent characterization of the selected sdAbs yielded binders to only one epitope, an additional sandwich panning scheme was carried out in an attempt to identify binders to additional epitopes which failed to be selected by the traditional panning methodology. In this method, sdAb H9 from the first panning protocol as well as mAb S222 were first immobilized as a capture antibody, followed by addition of SEB and then addition of the phage library as described in the methods section. Binders which compete with the immobilized panning antibody would only bind SEB released from the surface and be removed during washing; as a result, only phage displaying sdAbs that bind a unique epitope will be retained. Monoclonal ELISA of 48 colonies from each round of panning for each capture antibody resulted in over 85% positive binders each from the H9 and S222 captures. Five colonies from two rounds of panning for each capture antibody were sent for sequence analysis resulting in 20 unique sdAb sequences. Sequencing results revealed four were members of the same sequence family as B1, B12, and H9 from the initial panning sdAbs and these were not characterized further as they likely recognized the same epitope. Additionally, one sequence, S222-H4, was dissimilar to both families and later found not to bind SEB and was not characterized further. While the presence of these binders indicates that the sandwich panning approach failed to eliminate all binders to the epitope recognized by sdAbs isolated from conventional panning, the vast majority belonged to a unique sequence family. Thus, the approach was successful in identifying binders to a unique epitope. All of the other sequences were unique and comprised two distinct families with one containing ten members and one containing five. Three sdAbs from the larger family (H9-A1, H9-H11, and S222-A2) and two from the smaller family (H9-B11 and H9-D7) were characterized further (Figure 1). Three clones (H9-H11, S222-A2, and H9-B11) which produced poorly when transferred to the expression vector were modified to remove the hinge region which improved protein production from less than 1 mg/L to 5–10 mg/L.

### 2.4. Determination of Melting Temperatures and Refolding

**Figure 2.**
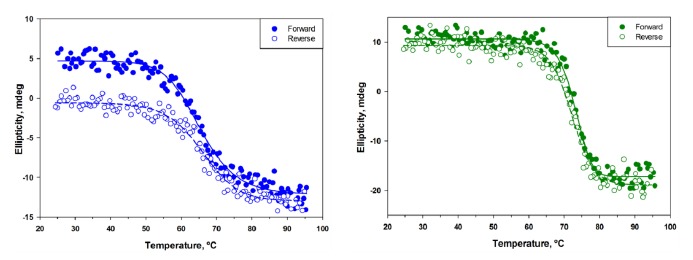
CD melting and refolding curves. Temperature scan performed from 25–90 °C in forward and reverse directions at a scan speed of 0.3 °C/min. Total refolding defined when ellipticity is completely recovered as demonstrated for B1 (**right**). Partial refolding defined as incomplete recovery of ellipticity value upon cooling as observed for E9 (**left**).

Two methods, circular dichroism spectroscopy (CD) and thermofluor assay (TFA), were employed for determination of melting temperatures of sdAbs. Melting temperatures (T_m_s) ranged from 60.0 to 72.0 °C as determined by CD and 55 to 71 °C by TFA (Table 1). CD melting curves were obtained both in the forward (heating) and reverse (cooling) direction to determine the degree of refolding of each of the sdAbs (Figure 2). Five of the antibodies (B12, F1, A10, B1, and H9) were observed to completely refold, based upon the ellipicity values obtained initially and after cooling. The remaining four sdAbs were observed to partially refold, and the percent refolded ranged from approximately 40% (E2) to 80% (H9-B11, H9-D7). The percentage of refolded protein was determined from the percentage of ellipticity recovered upon cooling.

### 2.5. Binding Affinity by SPR

**Figure 3.**
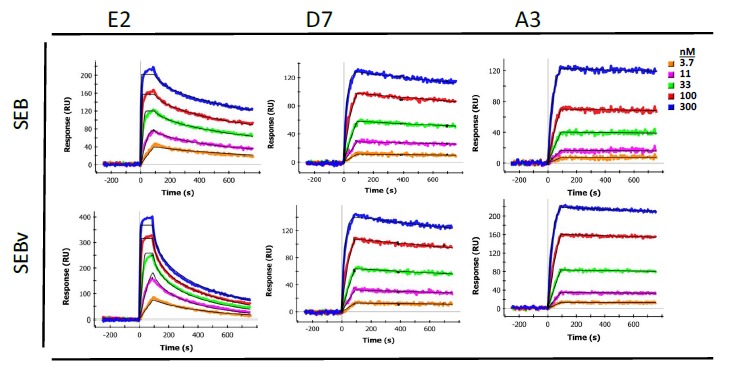
SPR traces for binding of sdAbs E2, D7, and A3 to immobilized SEB and SEBv. SEB and SEBv were immobilized onto gold SPR chip and sdAbs were flowed across chip at concentrations shown.

Binding affinity and kinetics of purified sdAbs towards SEB were determined by surface plasmon resonance (SPR) using a Bio-Rad ProteOn XPR36 system (Table 1). Purified sdAbs were flowed over a standard GLC chip upon which SEB was immobilized. Dissociation constants (K_D_) were all in the nanomolar range or lower, ranging from 4.16 × 10^−9^ M (S222-A2) to 9.0 × 10^−11^ M (H9). Association rate constants ranged from 1.54 × 10^5^ to 1.28 × 10^7^ M^−1^·s^−1^ and dissociation rate constants ranged from 1.92 × 10^−4^ to 5.06 × 10^−3^ s^−1^. Interestingly, all the initially selected sdAb had a lower K_D_ than those from the sandwich panning; these kinetic differences may be responsible for their segregation during the initial selection process. Additionally, binding of each sdAb was measured with both SEB and SEBv immobilized onto the SPR chip. A difference in binding kinetics was observed with SEB and SEBv (Figure 3) for all sdAbs identified in the initial panning, as shown for E2. Specifically, each of the sdAbs had a somewhat higher dissociation rate for SEBv compared to SEB. This difference was not evident in sdAbs identified by sandwich panning, as shown for D7 or A3 from the previous work [20]. The SPR was also utilized to evaluate binding competition of the sdAbs by first allowing one sdAb to bind in three of the six lanes, and then flowing three additional sdAb, each on a lane prebound by the first sdAb, and also on an unexposed lane to allow direct evaluation of competition. These results indicated that all the initial binders bound an overlapping epitope, and likewise all of the binders selected by the sandwich format also bound an overlapping epitope (not shown).

### 2.6. Direct Binding and Sandwich Assays of SEB

**Figure 4.**
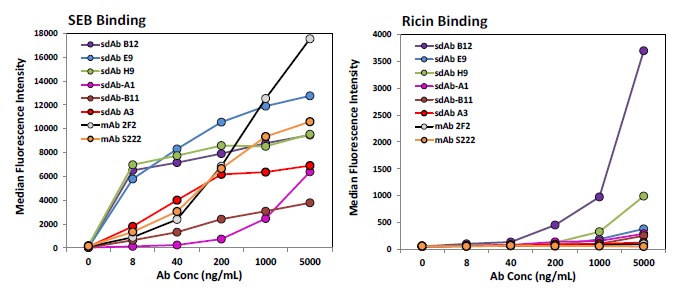
SEB direct-binding assay for selected sdAbs and mAbs (collectively termed Abs). SEB and ricin were cross-linked onto MagPlex microspheres. Biotinylated Abs were added at the concentrations shown. Fluorescence detection carried out upon addition of streptavidin-phycoerythrin. The error (SEM) was less than 10% for all values.

Direct binding to SEB was carried out via a MagPlex magnetic microspheres based assay to evaluate the biotinylation process and check for specificity of binding. Each of the sdAbs identified here as well as sdAb A3 from previous work [18] and two mAbs, 2F2 and S222, were tested. With the exception of sdAb B12, the sdAbs and the mAbs demonstrated good specificity showing low binding to additional protein coated bead sets included in the assay, binding to the ricin MagPlex bead set showed the most non-specific binding and is shown (Figure 4, right). Unexpectedly, in this format many of the current sdAbs exhibited a greater apparent affinity for SEB than A3 as well as the mAbs (Figure 4, left).

**Figure 5.**
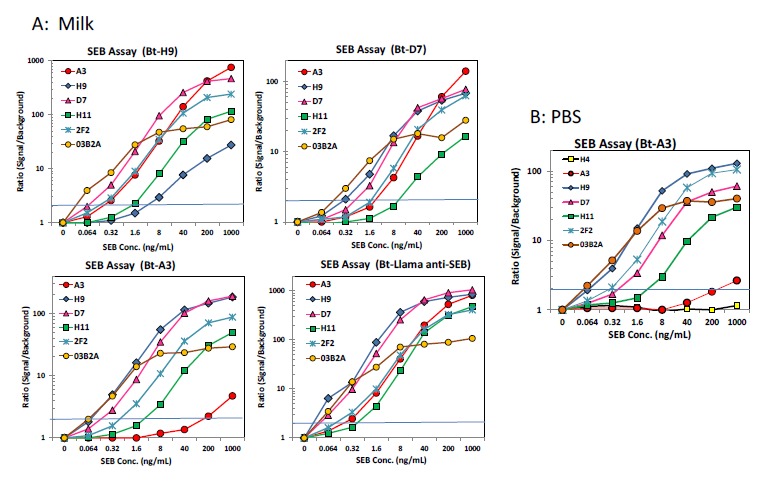
Sandwich-format MagPlex assay in milk. Capture beads contained each of the Abs cross-linked onto the surface as indicated. SEB was added at the concentrations shown followed by biotinylated (Bt) tracer Abs. Fluorescence detection carried out after addition of streptavidin-phycoerythrin. The error (SEM) was less than 10% for all values (not shown). B: MagPlex assay in PBS including a non-SEB binding sdAb capture set (H4).

Sandwich format MagPlex bead assays were performed in order to demonstrate the applicability of the sdAbs for the detection of SEB in complex matrices, as well as evaluate whether the sdAbs in this work bound to the same epitope as those from the previous work. Sandwich format assays were performed in both PBST (PBS buffer containing 0.05% v/v Tween-20) as well as milk spiked with various concentrations of SEB. Results of the sandwich-format MagPlex assays (Figure 5) indicated that all of the sdAbs isolated in the initial panning can be used in a sandwich assay with A3, mAb 2F2, as well as each of the sdAbs isolated by sandwich panning but not with one another. This confirmed that these sdAbs bind to the same unique epitope or overlapping epitopes. The sdAbs isolated by sandwich panning with mAb S222 and H9 also functioned in a sandwich assay format with A3, as well as mAb S222 and each of the sdAbs isolated in the initial panning of the library, suggesting that they bind to a unique additional epitope. These sdAbs did not function with one another, which likewise confirmed that they bind to the same epitope. The limit of detection varied between 64 and 320 pg/ml depending on the capture—detector pair being evaluated.

### 2.7. Epitope Mapping

**Figure 6.**
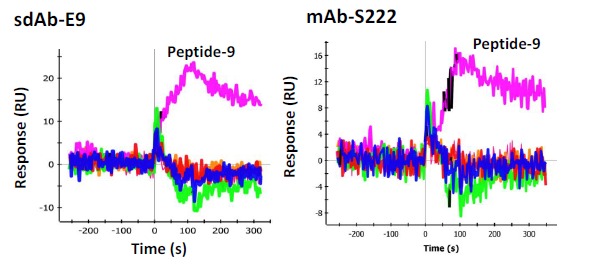
SPR of peptide fragments of SEB for epitope mapping.

**Figure 7.**
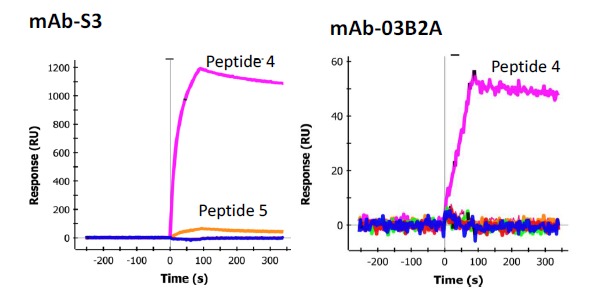
Binding of mAb-S3 and mAb-03B2A to selected peptide fragments of SEB. mAb-S3 generates a robust signal and is thought to recognize a denatured linear epitope.

In order to determine the epitope the sdAbs recognized, 33 individual synthetic biotinylated peptides spanning the entire length of SEB, 16-mers with an overlap of 8 (Genscript), were immobilized on separate spots on a GLC sensor chip. This was accomplished by sequentially coating the six lanes of the sensor chip with NeutrAvidin (NA) (Thermo) using the standard EDC chemistry. After each immobilization step the chip was turned 90 degrees and 6 of the 32 peptides were flowed over the chip surface for 5 min (30 uL/min) to immobilize the peptides to the NA spots, an average of 400 RU was observed. The chip was the returned to the original orientation to coat the next lane with NA. This process was repeated until all the peptides were immobilized to a specific spot. The cross immobilization was less than 10%. The remaining 3 spots were coated with peptides 1, 3, and 5 on the first chip tested and peptides 8, 9, and 10 on the second chip used for confirmation testing. To test the sdAbs, they were flowed across each spot and binding events were monitored. Binding was observed to peptide fragment 9 (VKSIDQFLYFDLIYSI) by sdAb E9 as well as mAb S222 (Figure 6). Another mAb, S3 was found to bind to peptide 4 (VLAESQPDPKPDELHK) very strongly (Figure 7, left panel), however it is thought to recognize a linear buried epitope as it binds to denatured SEBv more strongly than to the native protein (not shown). MAb 03B2A was also observed to bind to peptide 4 (Figure 7, right panel), but much more weakly, however it binds native SEB with high affinity (not shown).

## 3. Discussion

In earlier work, a single SEB-binding sdAb, A3, was identified [18]. Thus, part of the aim of this work was to characterize novel SEB binding sdAbs recognizing different epitopes such that they could be used in the development of sandwich-format assays with A3. Three immunodominant epitopes in SEB have been previously identified [21]. The epitopes bound by the sdAbs described here appear to differ from those generated in response to a recombinant SEB.

To generate sdAbs specific for SEB, we constructed a phage-display library based on mRNAs isolated from peripheral blood lymphocytes from a llamas immunized with either SEB toxoid or an SEBv vaccine. Evaluation of serum obtained from the immunized animals demonstrated a robust immune response in the animal immunized with SEB toxoid and a relatively diminished immune response in the animal immunized with the SEBv vaccine. This may have been due to differences in prior exposure, or differences in the immunogenicity of the proteins. The SEBv vaccine has been shown to be protective in mice, however, here the toxoid was more useful in generating new antibodies for immunoreagents [22].

Initial characterization of the thermal stability of the sdAbs displayed a range of melting temperatures (T_m_s) from approximately 60–73 °C. This range of melting temperatures, while not exceptional, is comparable to many other sdAbs. By comparison, the sdAb A3 from our previous work possessed an unusually high T_m_ of ∼83 °C. Thermal stability studies using the TFA technique confirmed a similar trend in T_m_s, although T_m_s measured by this method tended to be ∼2 °C less than those measured by CD. Exceptions to this trend were sdAbs D9 and H12, whose T_m_s as measured by TFA were ∼10 °C and 5 °C less than that measured by CD, respectively. This is likely a result of the different methods of monitoring the unfolding process by each technique. In CD measurements, loss of protein secondary structure is observed, while in TFA, the interaction of a dye to hydrophobic residues exposed upon unfolding is monitored. Melting curves obtained by CD in both heating and cooling directions additionally demonstrated that almost all of the sdAbs described in this work were able to either completely or partially refold upon cooling. Remarkably, 6 of the 14 sdAbs were able to completely refold and only one exhibited no refolding (Table 1).

Binding affinities determined by SPR for each of the initial sdAbs were in the sub-nanomolar range, and were comparable to that of A3 anti-SEB sdAb. Determination of association and dissociation rate constants revealed somewhat different binding kinetics when compared to A3. The association rate constants for each of the initially isolated sdAbs described here are on the order of 10^6^–10^7^ M^−1^·s^−1^, which is one-to-two orders of magnitude greater than that of A3. Conversely, the dissociation rate constants were also one-to-two orders of magnitude greater than that of A3, which has a remarkably low dissociation constant (Figure 3).

In an effort to identify SEB binders to yet another epitope, mAb S222 and H9 from the initial panning products were each used in a sandwich-format panning strategy on the original unpanned phage library. This method identified several new binders in novel sequence families, suggesting they may bind unique epitopes. These sdAbs had somewhat different CDR2s than the initial binders and much shorter CDR3s. Sandwich-format MagPlex assays confirmed that each of these sdAbs functioned in sandwich assays with each of the sdAbs from the initial panning. However, none worked with mAb 2F2 or with one another. These results confirmed that they all bind an overlapping epitope that is also recognized by mAb 2F2.

Upon inspection of the characteristics of the sdAbs isolated by the sandwich-format panning procedure, it was apparent that the identified sdAbs had on average lower melting temperatures as well as reduced on rates and overall affinities. Conversely, these sdAbs had more favorable off rates. While it is likely these sdAbs were out-competed in the initial conventional panning protocol due to their slow on rates, their low off rates make them very amenable to the development of detection assays. The observed decrease in Tm is likely a happenstance as this property was not under selective pressure. Use of a sandwich-format panning protocol as described in this work was successful in identifying binders to a new epitope which would have otherwise been lost due to their relatively lower affinity. Binders to different epitopes are critical for many applications [23].

Since one of our motivations in identifying novel SEB-binding sdAbs was to develop a pair of sdAbs which could be used in a sandwich assay format for the detection of SEB, we evaluated each of our new sdAbs in addition to our previously-identified sdAb A3 and mAbs 2F2 and S222 in a sandwich format on MagPlex magnetic microspheres in both PBST and milk spiked with SEB. This format afforded us the ability of simultaneously measuring the interaction of each of our sdAbs as capture antibodies crosslinked onto the microspheres against biotinylated tracers which included all of our newly identified sdAbs as well as A3, 2F2 and S222. This highly-multiplexed, competition-based immunofluorescence format allowed us to conveniently determine which pairs of antibodies were able to function in a sandwich format. Each of the sdAbs identified in the initial panning functioned well with both A3 and 2F2 as tracers (Figure 5). Conversely, none of the initially selected sdAbs were successful as a capture antibody with S222 or with one another. This suggests that these sdAbs and S222 recognize a similar epitope on SEB, unique from the epitope recognized by A3 and 2F2.

In an effort to determine the specific epitope recognized by the sdAbs, 33 peptides were synthesized that spanned the sequence of the SEB toxin. Each peptide, which overlapped in sequence by eight residues, was synthesized with an attached biotin to provide convenient immobilization to a NeutrAvidin coated SPR chip which had been prepared serially for this application. Each peptide was immobilized on a unique spot on the surface of a chip and then anti-SEB sdAb and mAbs were flowed over the peptides. One peptide exhibited binding by the initially selected sdAbs, E9 in particular. The sequence of this peptide, VKSIDQFLYFDLIYSI, includes L45 (underlined), a position known to be important for the binding of the SEB to the major histocompatibility complex (MHC). Interestingly, mutation of L45 to arginine results in a less toxic variant of SEB unable to bind MHC. Indeed L45R is among the three changes present in the SEBv variant, a non-toxic triple mutant of SEB that has previously been developed by researchers as a vaccine [19].

Since the epitope recognized by the first set of sdAbs contains the L45 position, we hypothesized there might be a difference in affinity between SEB and the non-toxic SEBv. Indeed, all of the initially selected binders showed a clear difference in binding to the two variants as typified by E2, shown in Figure 3. Interestingly, mAb S222 which also binds the same epitope and peptide did not show a differential affinity, suggesting its binding interactions with SEB is distinct from the sdAbs. The sdAbs isolated second were not observed to bind any of the peptides on the SPR chip, thus their epitope was not defined.

## 4. Conclusions

We have isolated and characterized fourteen sdAbs specific to SEB. Each of these sdAbs exhibited nanomolar or lower dissociation constants. Additionally, melting temperatures ranged from around 60 °C to over 70 °C and all but one demonstrated some amount of refolding after denaturation; six were able to completely refold after thermal denaturation. Epitope mapping and competitive sandwich-format assays suggested that all of the sdAbs isolated through a conventional panning protocol bound to the same epitope in SEB (VKSIDQFLYFDLIYSI) containing position L45, which is located in a portion of the toxin involved in binding to the MHC. Further characterization of the binding of the sdAbs confirmed that several of the sdAbs selected from the direct panning, which bind an overlapping epitope, show different binding kinetics to SEB and non-toxic variant containing an L45R mutation known as SEBv. A second set of sdAbs isolated from a sandwich panning strategy all bound to a second unique epitope. We also demonstrated that our newly isolated sdAbs can function in a sandwich format assays in a complex matrix (low fat milk) with a previously described SEB-binding sdAb, A3, as well as mAbs 03B2A, S222, and 2F2 with good sensitivity. Thus, we now have the ability to detect SEB using an all sdAb reagent containing assay with limits of detection as low as 64 pg/mL, in many cases as good as those obtained using conventional antibodies.

## 5. Materials and Methods

### 5.1. Source of Antibodies and Other Chemicals

Except for where stated otherwise, reagents and buffers were obtained from Sigma-Aldrich (St. Louis, MO, USA) or VWR International (Radnor, PA, USA). Restriction endonucleases and T4 DNA ligase were obtained from New England Biolabs (Ipswich, MA, USA). DNA sequencing services were provided by Eurofins MWG Operon (Huntsville, AL, USA). MAb 2F2 was the kind gift of Tom Obrien (Tetracore, (Rockville, MD, USA)). MAb S222 and S3 were the kind gifts of Peter Sevesnikov (Russian Research Center for Molecular and Diagnostics and Therapy,Moscow, Russia). The mAb 03B2A was the kind gift of Jill Czarnecki (Naval Medical Research Center, (Bethesda, MD, USA)).

### 5.2. Immunization Protocol

Two llama were immunized with 5 doses of antigen (100 μg/injection), once every three weeks, and a full bleed was completed two weeks after the final injection. The first llama, Centavo, was immunized with SEB Toxoid (Toxin Technologies, (Sarasota, FL, USA) and the second, Whisper, with recombinant inactive SEBv vaccine (BEI Resources, Manassas, VA, USA). SEBv vaccine was obtained through the NIH Biodefense and Emerging Infections Research Resources Repository, NIAID, NIH: Staphylococcal Enterotoxin B Toxoid, Recombinant from *Escherichia coli*, NR-10049. Immunizations and bleeds were administered by Triple J Farms (Bellingham, WA, USA) and approved by the Institutional Animal Care and Use Committee (IACUC).

### 5.3. Library Preparation

After immunization, blood was drawn from each animal and peripheral blood lymphocytes were purified using Uni-SEP maxi+ tubes (Novamed, Jerusalem, Israel). Cellular RNA was purified from isolated lymphocytes using the QIAamp RNA Blood Mini Kit (Qiagen, Hilden, Germany) from purified RNA, a cDNA library was constructed using the SuperScript II Rt-PCR kit (Invitrogen, Grand Island, NY, USA). From this cDNA library, genes encoding for sdAbs were amplified by PCR using primers with corresponding to sequences which flank the sdAb variable domain as described by Ghahroudi [24]. The sdAb gene library was then inserted into the SfiI restriction site of phage display vector, pECAN21 and transformed into electrocompetent XL-1 Blue cells (Stratagene, La Jolla, CA, USA) [25]. Direct colony counting of transformants following cloning was used to determine library size and diversity was determined by sequencing 20 individual transformants.

### 5.4. Phage Library Panning

The phage library was subjected to two rounds of panning using SEB adsorbed onto the surface of wells of a Nunc Maxisorp plate following a protocol described previously. Briefly, 100 μL of SEB was deposited overnight in four wells at a concentration of 5 μg/mL in phosphate buffered saline (PBS). After triplicate washing with PBST, wells were blocked with PBST containing 5% milk powder (w/v) (MPBST) for two hours at room temperature and then washing was repeated. Next, 75 μL of the phage library and 25 μL of MPBST was added to each well and incubated at room temperature for two hours. Following the phage binding step, the wells were thoroughly washed. In round one, 20 rounds of washing with PBST followed by PBS were performed. In round two, stringency was increased by performing 40 rounds of washing with each. Bound phage was eluted from each well in 100 μL of 100 mM triethylamine incubated at room temperature for 10 min and then neutralized in 400 μL 1 M tris(hydroxymethyl)aminomethane (TRIS). Eluted phage from each round was used to infect a 10 mL culture of XL-1 Blue cells (OD_600_ = 0.5) and the titer of both the input phage stock and output phage was determined by culture dilution and direct colony counting. For the sandwich panning protocol, wells were treated with a solution of either sdAb H9 or mAb S222 at a concentration of 5 μg/mL in PBS followed by SEB as described above. Remaining steps in the panning procedure were carried out as described above.

Following each round of selection, polyclonal phage ELISA was performed to evaluate the quality of each round. SEB was adsorbed into wells of a 96-well Nunc Maxisorp by adding 100 μL of 5 mg/mL SEB to each well and incubating overnight at 4 °C. Excess SEB was removed, and the wells were washed in triplicate by PBST and PBS. Wells were blocked for 2 h at room temperature with MPBST and then washed again as before. To triplicate wells, 1 × 10^10^ phage was added and allowed to bind for 90 min at room temperature, and then washing was repeated. The ELISA was developed by incubating wells with 100 μL SureBlue substrate solution (KPL Laboratories, Gaithersburg, MD, USA) for 10 min followed by the addition of 50 μL of 1 M sulfuric acid. The ELISA signal was determined by subtracting Abs 450 nm from Abs 650 nm as measured on a Tecan Infinite M100 fluorescence plate reader (Tecan Group Ltd., San Jose, CA, USA). Monoclonal phage ELISA was utilized to select individual binders for evaluation by DNA sequencing, and was performed in a similar procedure, except phage amplified from individual colonies from each round of panning was used in place of amplified polyclonal phage in the protocol above.

### 5.5. Cloning, Expression, and Purification

Plasmid from individual colonies of positive SEB binders identified by monoclonal phage ELISA was purified from overnight cultures using the QIAprep Miniprep Kit (Qiagen) and sequenced by Eurofins MWG Operon (www.operon.com). Genes coding for individual sdAbs from the isolated plasmids were cloned into expression vector pECAN45 by restriction digestion with SfiI and ligated with T4 DNA ligase. Expression plasmids were maintained in Top10 cells (Life Technologies, Grand Island, NY, USA) and transformed into Rosetta (DE3) cells (Novagen) for protein expression. In order to improve the yield of three of the sdAbs (S222-A2, H9-B11, and H9-H11), the genes were amplified by PCR to remove the C-terminal hinge region and cloned into the NcoI and NotI sites of expression vector pET22b. Expression and purification of individual sdAbs was performed as described previously [25]. Purification of sdAbs isolated from the periplasmic space was accomplished by immobilized metal affinity chromatography (IMAC) followed by FPLC size exclusion chromatography using a BioLogic Duo Flow FPLC system (BioRad, Hercules, CA, USA) equipped with a Sephadex G75 column. The concentration of purified protein solutions was determined on a NanoDrop 2000 spectrophotometer (Thermo Scientific, Waltham, MA, USA).

### 5.6. Determination of Protein Thermal Stability and Refolding by Circular Dichroism (CD)

The melting temperature (T_m_) and refolding of each sdAb was determined as previously described using a Jasco J-815 CD Spectropolarimeter (Easton, MD, USA) equipped with a PTC-423S Peltier temperature controller [26,27]. Purified sdAbs were diluted to a concentration of 0.02 mg/mL in water. CD measurements were performed in a 1.0 cm quartz cuvette and ellipticity was monitored at 205 nm as the temperature was scanned from 25 °C to 95 °C at a rate of 2.5 °C/min. The data pitch was 1 nm, D.I.T was 8 s, and bandwidth was 1.0 nm. To monitor refolding, ellipticity was also monitored while the temperature was scanned from 95 °C to 25 °C as well, with the same parameters.

### 5.7. Determination of Protein Thermal Stability by Thermofluor Assay (TFA)

The T_m_ of each sdAb was also confirmed by TFA. This technique relies on the fluorescence enhancement of a dye, Sypro Orange (Sigma), as it interacts with the hydrophobic amino acids on a protein as they become accessible upon thermal unfolding. Each sdAb was measured at a concentration of 500 μg/mL in a total volume of 20 μL PBS. Sypro orange dye (Sigma) was added at a dilution of 1:1000. Samples were measured in triplicate using a StepOne Real-time PCR machine (Applied Biosystems, San Franciscto, CA, USA). The heating program was run in continuous mode from 25–99 °C at a heating rate of 2 °C/min. Data was recorded using the ROX filter and the melting point was determined to be the peak of the first derivative of the fluorescence intensity.

### 5.8. Binding Affinity and Kinetics Determination by Surface Plasmon Resonance (SPR)

SPR affinity and kinetics measurements were performed as described previously using the ProteOn XPR36 (Bio-Rad) [20]. A GLC chip was coated with SEB and SEBv. Immobilization was performed using proteins diluted in 10 mM acetate buffer pH 5.0 and attached to the chip following the standard EDC coupling chemistry available from the manufacturer. Binding kinetics of each sdAb were tested at 25 °C by flowing six concentrations varying from 1000 to 0 nM at 100 μL/min for 120 s over the antigen coated chip and then monitoring dissociation for 600 s. Following each run, the chip was regenerated by flowing 50 mM glycine-HCl (pH 2.5) with 0.05% SDS across the surface for 36 s. Data analysis was performed with ProteOn Manager 2.1 software, corrected by subtraction of the zero antibody concentration column as well as interspot correction. Binding constants were determined using the Langmuir model built into the analysis software.

### 5.9. MAGPIX Immunoassay Direct Binding Analysis

Direct binding assays of each sdAb was completed as described previously [27,28]. For the direct binding experiments, SEB was crosslinked to carboxylated magnetic microspheres (Luminex, Austin, TX, USA) via a two-step carbodiimide coupling protocol as detailed by the manufacturer. In wells of a microtiter plate, biotinylated sdAbs (Bt-sdAbs) were added in the top wells of the plate and then serial diluted down each column using PBST. The final row contained only PBST as a blank. To these wells, SEB-coated beads were added (5.0 μL per well) and incubated at room temperature for 30 min. Unreacted Bt-sdAbs were removed by washing and then 50 μL/well of streptavidin-phycoerythrin (SA-PE, 2.5 mg/L) was added to each well. Samples were measured using MAGPIX (Luminex). Results were reported as the median fluorescence intensity of at least 50 individual microspheres.

### 5.10. Multiplexed MAGPIX-Based Sandwich Format Assay

For sandwich format assays, sdAbs were crosslinked to MagPlex magnetic microspheres (Luminex) using carbodiimide chemistry described above, similar to as described previously [20,28]. In the top row of wells in a microtiter plate, 100 μL of 1.0 μg/mL SEB (Staphylococcal Enterotoxin B, Purified from *Staphylococcus aureus* subsp. *aureus*, NR-861 was obtained through the NIH Biodefense and Emerging Infections Research Resources Repository, NIAID, NIH) in PBST was added, and serial dilutions were made down each column with the bottom row containing only PBST as a blank. To each well, a cocktail containing an aliquot of each of the sdAb-crosslinked microspheres was added and incubated at room temperature in the dark for 1 h. To wells in separate columns, Bt-sdAbs along with Bt-mAbs 2F2 and S222 were added to a final concentration of 1.0 μg/mL and incubated 20 min at room temperature in the dark. Following incubation, the microspheres were washed thoroughly with PBST. As above, SA-PE was added to all wells, and samples were measured using MAGPIX.

### 5.11. Epitope Mapping by SPR

To epitope map the binding site of SEB for these sdAbs a new method was developed that utilized the ProteOn XPR36. 33 overlapping biotin labeled peptides (16-mers, with an overlap of 8) were purchased from Genscript (Piscataway, NJ, USA). The peptides were dissolved in DMSO. To prepare a peptide coated sensor chip, a GLC chip was coated serially one column at a time with NeutrAvidin using the standard EDC/sulfo-NHS coupling protocol provided by the manufacturer. The orientation of the chip was changed after each immobilization step and that NeutrAvidin column was coated with six of the biotin labeled peptides (∼1–4 ug/mL in PBST). After the 33 different peptides were immobilized the remaining 3 spots were coated with peptides 1, 3, and 5 on the first chip prepared. A second chip was prepared in order to confirm the results of the first chip, and on that chip the additional spots were coated with peptides, 8, 9, and 10.

## Figures and Tables

**Table 1.** Melting temperature determination, refolding ability, and binding kinetics for SEB-binding sdAbs.

sdAb	Refold					
	T_m_(CD), °C	T_m_(TFA), °C	(%, approx.)	K_a_(1/Ms)	K_d_(1/s)	K_D_(M)
B12	65.3	64	100	1.39 × 10^6^	3.21 × 10^−4^	2.32 × 10^−10^
F1	70.9	68	100	6.46 × 10^5^	4.20 × 10^−4^	6.50 × 10^−10^
A10	65.5	63	100	1.76 × 10^6^	2.77 × 10^−4^	1.58 × 10^−10^
D9	64.6	55	60	2.49 × 10^6^	2.77 × 10^−4^	1.11 × 10^−10^
B1	73	71	100	1.95 × 10^6^	3.90 × 10^−4^	2.0 × 10^−10^
H9	70.9	70	100	3.53 × 10^6^	3.18 × 10^−4^	9.0 × 10^−11^
H12	60	55	40	2.23 × 10^6^	6.07 × 10^−4^	2.72 × 10^−10^
E9	65.1	62	70	1.11 × 10^6^	5.89 × 10^−4^	5.33 × 10^−10^
E2	72	71	60	1.28 × 10^7^	3.51 × 10^−3^	2.73 × 10^−10^
H9-A1	66.9	*	60	1.8 × 10^6^	2.27 × 10^−3^	1.25 × 10^−9^
H9-H11	60.4	59	0	4.85 × 10^5^	7.55 × 10^−4^	1.56 × 10^−9^
S222-A2	56.2	55	100	1.22 × 10^6^	5.06 × 10^−3^	4.16 × 10^−9^
H9-B11	58.5	60	80	1.54 × 10^5^	5.12 × 10^−4^	3.32 × 10^−9^
H9-D7	62.1	61	80	1.74 × 10^5^	1.92 × 10^−4^	1.1 × 10^−9^
